# Evaluation of the reliability of the EuroSCORE riskanalysis prediction in high-risk older patients undergoing CABG

**Published:** 2009-12

**Authors:** Hïkmet Iyem

**Affiliations:** Department of Cardiovascular Surgery, Dicle Universty, Diyarbakır, Turkey

## Abstract

**Background:**

The aim of this prospective study was to evaluate the reliability of EuroSCORE risk-analysis predictions on early mortality in high-risk older patients who underwent heart surgery.

**Methods:**

From January 2008 to February 2009, a total of 128 consecutive high-risk older patients who underwent open-heart surgery were included. Patients who required emergency surgery, had pulmonary hypertension, a recent myocardial infarction, underwent combined heart surgery procedures or had renal disease were included. The patients had a mean age of 72 ± 9 years (range 64–91, 53.1% male) and were evaluated for surgery.

**Results:**

Coronary artery bypass graft (CABG) surgery was performed on 112 patients and valve surgery on 16. Eight patients (6.25%) died in hospital. The observed mortality rate was lower than the expected mortality obtained using EuroSCORE (6.25% vs 11.2 ± 7.2%, respectively, *p* < 0.021).

**Conclusion:**

There was no correlation between the pre-operative logarithmic score of expected mortality and the observed mortality rate in these older high-risk patients who underwent open-heart surgery.

## Summary

The analysis of patient outcome is gaining increasing importance as public and health authorities demand more data on risk, prognosis and performance of specific procedures, particularly for resource-intense operations such as coronary artery bypass graft (CABG) and heart valve surgery. It also helps the patient as well as the family to weigh the risks and benefits, so as to clarify expectations.^1^

The growing interest in risk-adjusted analysis of outcome in cardiac surgery has led to the development and validation of several predictive models for postoperative mortality, morbidity, and prolonged hospital stay.^2^ Various risk-stratification systems have been developed in the last decade, the validity of which have been widely accepted by clinicians throughout the world.

The European system for cardiac operative risk evaluation (EuroSCORE) is a risk-stratification tool for peri-operative mortality after cardiothoracic surgery that was designed and developed in Europe and validated in North America. One additional risk point is added per five years of age over 60 years.^3^ It was developed with data collected from 130 centres in eight European countries. It recruited around 20 000 patients and was first published in 1999.^4^

It has been accepted that operative and in-hospital mortality are an indicator of quality of life in patients who have undergone heart surgery. The outcomes of patients undergoing heart surgery have therefore been reported annually and heart surgery centres have been classified according to mortality and morbidity rates. It is important to use scoring sytems to distinguish high-risk patients undergoing heart surgery otherwise the succes rates of some centres would be affected negatively by this patient population. It would mean low-risk patients would be addmitted to centres with low mortality rates, and high-risk patients would not undergo heart surgery in these centres. This situation therefore affects high-risk patients adversely, as they require much more care during and after the operation.^5^

The aim of this prospective study was to evaluate the reliability of EuroSCORE predictions on early mortality in older high-risk patients who underwent heart surgery.

## Methods

In this study from January 2008 to February 2009, a total of 128 consecutive high-risk older patients who underwent open-heart surgery were included. The EuroSCORE of all patients (standard and logarithmic values) was calculated before heart surgery (Table 1). Patients who required emergency surgery, had pulmonary hypertension, a recent myocardial infarction, underwent a combined heart surgery procedure or had renal disease were included. However, young and low-risk patients were excluded.

**Table 1:** Euroscore Risk Scoring

*Variables*	*Definition*	*Points*
Age (years)	> 60 years add 1 point for every 5 years	1
Gender	Female	1
Chronic pulmonary disease	Long-term use of bronchodilators or steroids for lung disease	1
Extracardiac arteriopathy	One or more of claudication, carotid artery occlusion or > 50% stenosis, previous or planned intervention on the abdominal aorta, limb arteries or carotids	2
Neurological dysfunction	Disease severely affecting ambulation or day-to-day functioning	2
Previous cardiac surgery	Requiring open pericardium	3
Creatinine > 200 μmol/l	Pre-operative > 200 μmol/l	2
Active endocarditis	Patient still on antibiotic treatment for endocarditis at time of surgery	3
Critical pre-operative state	Ventricular tachycardia/ventricular fibrillation or aborted sudden death, pre-operative cardiac massage, preoperative ventilation before anaesthetia, pre-operative inotropes or IABP, preoperative acute renal failure (anuria or oliguria < 10 ml/hr)	3
Unstable angina	Rest angina requiring i.v. nitrates until arrival in operating room	2
LV function	Moderate or LVEF 30–50%	1
	Poor or LVEF < 30%	3
Recent MI	Myocardial infarction within 90 days	2
Pulmonary hypertension	Systolic pulmonary artery pressure > 60 mmHg	2
Emergency	Operation before start of next working day	2
Other than isolated CABG	CABG and additional or major cardiac surgery	2
Surgery on thoracic aorta	Ascending arch or descending aortic diseases	3
Post-infarct septal rupture		4

Early mortality was defined as death within 30 days after the heart surgery. Patients were divided into three groups according to the EuroSCORE risk:^3^ low-risk patients (≤ 2 points), patients at moderate risk (3–5 points) and high-risk patients (≥ 5 points). The total score was calculated and compared with the EuroSCORE value in each group. The predictive factors affecting the success of the operation, such as age, gender, peripheral arterial disease, chronic obstructive pulmonary disease, renal disease and recent cerebrovascular disease are shown in Table 2. An informed, written consent was obtained from all patients, who were operated on by the same surgeon.

**Table 2:** The Pre-Operative Demographic And Clinical Parameters Of Patients

*Parameters*	*Values (%)*
Number of patients (*n*)	128
Female/male (*n*)	62/66
Age (years)	72 ± 9
Body weight (kg)	64.6 ± 17.4
Height (cm)	162.3 ± 5.6
Body mass index (kg/m^2^)	22.1 ± 3.2
Emergency (*n*)	18 (14.06)
Ejection fraction (%) (mean): moderate	38.6 ± 3.4
poor	27.7 ± 2.7
Recent myocadial infarction (*n*)	16 (12.50)
Left ventricular dysfunction (*n*)	31 (24.21)
Serious arrhythmias (*n*)	12 (9.37)
Chronic obstructive pulmonary disease (*n*)	43 (33.59)
Diabetes (*n*)	77 (60.15)
Smokers (*n*)	81 (63.28)
Chronic renal failure (*n*)	11 (8.59)
Peripheral arterial disease (*n*)	17 (13.28)
Craniocervical vascular disease (*n*)	6 (4.68)
Valvular disease (*n*)	16 (12.50)
Coronary artery disease: on CABG pump (*n*)	112 (87.5)
Re-operation (*n*): coronary	0 (0)
valvular	2 (2.94)

## Anaesthesia

In the operating room, ECG monitoring and radial artery cannulation for systemic arterial blood pressure monitoring were done. A peripheral venous line, central jugular vein catheters and urethral catheters were inserted. Body temperature was monitored with rectal and oesophageal probes. The usual protocol of our cardiovascular surgery clinic was applied to all patients. All routine cardiac medications were continued until the morning of surgery.

Anesthesia was induced with 20 mg/kg fentanyl citrate, 0.12 mg/kg pancuronium and 2 mg/kg propofol. After tracheal intubation, mechanical ventilation was institued with 6–8 ml/kg tidal volume and 10–12/min respiratory rates. Anesthesia was maintained with 10 mg/kg of fentanyl and 1 mg/kg propofol as infusion, and 2 mg of pancuronium were given every 45 minutes throughout the operation.

## Operative technique

A standard median sternotomy, aorta–right atrial cannulation, and cardiopulmonary bypass were performed on the on-pump patients. During the cardiopulmonary bypass, haematocrit, mean arterial pressure and pump flow were maintained at 25–30%, 50–70 mmHg and 2.2–2.5 l/m2, respectively. Tissue perfusion, urine output and base deficit were monitored for adequacy. Patients were cooled to 30°C with moderate hypothermia. Desflurane–remifentanil anesthesia was administered during cardiopulmonary bypass. Revascularisation procedures were performed with aortic cross clamping. During myocardial ischaemia, antegrade cold, hyperkalaemic blood cardioplegia was used. After completion of the distal anastomosis, the proximal anastomosis was performed to the ascending aorta using a side-biting clamp.

All patients recieved pericardial (no. 28) and mediastinal (no. 32) chest tubes. In the open-pleura group, additional left hemithorax (no. 32) tubes were also placed for drainage, and removed routinely on the first postoperative day, upon change in the drainage towards serosity.

## Statistical analysis

All statistical tests were performed using the statistical analysis program SPSS 11.0 for Windows. Demographic results are expressed as mean value ± standard deviation. The Student’s *t*-test and χ^2^-test, respectively, were performed to evaluate the analysis of continuous variables, which were compared with percentages. The relationship between risk factors and outcome was assessed by EuroSCORE logarithmic regression analysis with added risk factors. Furthermore, the difference between expected and observed mortality was tested with the Fisher’s exact test. A *p*-value < 0.05 was considered statistically significant.

## Results

The 128 patients were evaluated, and coronary bypass surgery was performed on 112 patients and valve surgery on 16 (aortic valve on nine and mitral valve on seven). No statistically significant difference was found between the CABG and valve surgery groups with regard to the drainage volume, extubation time, duration of stay in intensive care unit and duration of hospitalisation.

Eighteen patients had emergency operations for acute mitral insufficiency, due to myocardial ischaemia in two patients, unstable angina pectoris in 10, atherosclerosis of the left main coronary artery in four and endocarditis in two patients. Pre-operative and postoperative data of all patients are shown in Table 3. Eight patients (6.25%) died in hospital, six (4.68%) in the CABG group and two (1.55%) in the valve surgery group. The cause of death was low cardiac output in three patients, sepsis due to pulmonary infection in two, intracranial bleeding in two, and gastrointestinal bleeding in one.

**Table 3:** The Pre-Operative And Postoperative Paramaters Of Patients

*Pre-operative parameters*	*Values (%)*
Left internal mammary artery (*n*)	112 (87.5)
Valvular disease (*n*): aortic	9 (7.03)
mitral	7 (5.46)
Graft: on CABG pump (*n*)	2.2 ± 0.46
Cardiopulmonary bypass (min)	88.6 ± 4.4
Cross clamp (min)	36.5 ± 2.7
Need for inotropic agents (*n*)	43 (33.59)
*Postoperative data*	
Low cardiac output (*n*)	22 (17.18)
Intra-aortic balloon pump usage (*n*)	11 (8.59)
Drainage (ml)	520.6 ± 87.1
Blood transfusion (ml)	488.6 ± 93.4
Mechanical ventilation (hours)	14.6 ± 6.3
Intensive care unit stay (days)	1.3 ± 0.3
Hospital stay (days)	8.6 ± 1.27

An intra-aortic balloon pump was required in 11 patients due to low cardiac output in the post-operative period. Of these, two patients had left main coronary artery disease and nine had unstable angina. All complications related to the surgical procedure in the intra- and post-operative periods are shown in Table 4.

**Table 4:** Postoperative Complications

*Complications*	*n (%)*
Acute renal failure	14 (10.93)
Low cardiac output	11 (8.59)
Arrhythmia	18 (14.06)
Mesenteric ischaemia	2 (1.56)
Pancreatitis	2 (1.56)
Gastrointestinal bleeding	2 (1.56)
Stroke	3 (2.34)
Transient neurological deficit	5 (3.90)
Pulmonary complications	11 (8.59)
Re-exploration for bleeding	3 (2.34)
Wound infection	5 (3.90)
Sepsis	2 (1.56)
Mixed complications	6 (4.6)

The mean standard risk determined using the EuroSCORE database of logarithmic scores for total expected mortality was 11.2 ± 7.2%. However, the observed patient mortality rate was lower than the expected rate obtained with EuroSCORE (6.25% vs 11.2 ± 7.2%, respectively, *p* < 0.05) (Table 5).

**Table 5:** Expected And Observed Mortality Of Cardiac Surgical Patients

*Surgery*		*Expected EuroSCORE mortality*		*Observed mortality (%)*	
		*Standard risk value*	*Log value (%)*		
CABG	6	6.42 ± 3.5	10.8 ± 12.5	4.68	< 0.05
Heart valve	2	5.62 ± 2.1	11.5 ± 13.7	1.55	< 0.05
Total	8	6.20 ± 3.2	11.2 ± 7.2	6.25	< 0.05

## Discussion

A major problem in contemporary cardiac surgery and anesthesia is quantification of operative risk. Mortality, morbidity and the patient’s comfort are the most valuable parameters in evaluating the results and success rate of the surgical procedure. Mortality has been the most important factor indicating the success rate of heart surgery.

Recently however, the success rates of surgery have not been evaluated by means of mortality rates only. Scoring systems are now used to predict the risk of surgery at the pre-operative stage and the surgeon can easily perform these to estimate operative risk. The surgical strategy can therefore be determined by analysis of pre-operative risk factors prior to surgery, and the outcomes of surgery can thus be predicted.^3^

There are several scoring systems evaluating expected mortality rates and outcomes in patients undergoing heart surgery.^6^ The Parsonnet scoring system was the first, developed to evaluate pre-operative risk, and it was performed to predict expected mortality in 3 500 consecutive patients who underwent heart surgery in 1989.^6^ The Clevend Clinic scoring system was developed in 1992 to evaluate the risk stratification of mortality and morbidity.^7^ The Society of Thoracic Surgeons’ national database (STS) scoring system was pioneered on 728 consecutive patients and its results were reported in 1994.^8^ The Pons scoring system was developed in 1995.^9^ The French system (1995) was devised to evaluate expected mortality and morbidity in 7 181 consecutive patients undergoing heart surgery. In 1997, the Ontario Province risk score was devised.^10^ EuroSCORE is the most recent scoring system to determine expected mortality in a prospective study including 19 030 consecutive patients undergoing heart surgery.^11^

Mortality within 30 days is an extremely important indicator of a surgeon’s success rate and the quality of hospital service in open-heart surgery. Gissler *et al.* compared six different risk-scoring systems in a prospective study including 504 patients. They reported the EuroSCORE scoring system showed the most conformity between expected and observed mortality rates.^11^ In a study reported by Nashef *et al*,^5^ the EuroSCORE was recommended as a simple, objective scoring system to determine expected and observed mortality rates.

In the present study, the mean observed mortality rate was found to be 6.25%, whereas the expected rate calculated from EuroSCORE was 11.2 ± 7.2%. Several heart operations have been evaluated for early and late survival and in 1997, Sergeant *et al*.^12^ reported that EuroSCORE was lower in the high-risk group of patients and higher in the low-risk group in a study including 2 051 patients who underwent primary or redo coronary artery bypass surgery.

EuroSCORE is being used more frequently, as it is less complex than other systems and originated within Europe, with the participation of several Spanish hospitals. EuroSCORE is now being evaluated in the USA. Nevertheless, its diagnostic accuracy has not been clearly ascertained.^13^ The EuroSCORE description has three risk groups, based on the score obtained: low-risk patients (≤ 2 points) with a predicted mortality below 1%, patients at moderate risk (mortality around 3%), and a high-risk group (predicted mortality of 10–11%).^14^ In our study, there were no patients at low or moderate risk; all had a high risk (11.2 ± 7.2%) determined by the EuroSCORE risk analysis.

To date, the relationship between high pre-operative risk and quality of life (QOL) after CABG has often been studied in older individuals, who already have a higher risk due to co-morbidity and age. Nowadays, there is an increase in the number of older patients who undergo CABG.^15^ They usually have more extensive coronary artery disease and a worse total pre-operative state. Many reports show that older patients (with acceptable operative risk) have most benefits after CABG compared to their initial quality of life.^16^ This is concordant with our results that show a decline in the mortality after CABG in patients with a high EuroSCORE. However, some reports show that older persons tend to have less improvement after CABG, compared with younger patients.^17^ Radovanovic *et al.* examined the relationship between QOL and expected operative risk of open-heart intervention, defined by Parsonnet’s method. They found a negative correlation between the level of operative risk and the average pre-operative QOL index.^18^

Study limitations in the present study were that 68 patients were not enough to evaluate the efficacy of EuroSCORE in older, high-risk patients who underwent heart surgery, and the relationship between the QOL and operative risk were not assessed.

## Conclusion

There was no correlation between the pre-operative logarithmic score of expected mortality and the observed mortality rate in older high-risk patients who underwent open-heart surgery. Our study group included only high-risk older patients. As far as we know, there is no scoring system evaluating expected mortality in this particular population. The observed mortality rate changes according to the experience of the surgeon and clinic. With the advances in open-heart surgery, mortality in high-risk older patients has decreased, therefore the expected mortality rate calculated using EuroSCORE may be an overestimation in this patient group. The EuroSCORE system should therefore be modified for high-risk older patients.

Because our study group was small, we could not conclude that EuroSCORE was unreliable in predicting postoperative mortality. We still use this scoring system in our clinic for all patients undergoing open-heart surgery to determine their expected mortality. However, we need larger studies to evaluate the reliability of EuroSCORE in this high-risk patient population.
